# Dispatcher-assisted cardiopulmonary resuscitation for traumatic patients with out-of-hospital cardiac arrest

**DOI:** 10.1186/s13049-019-0679-2

**Published:** 2019-11-01

**Authors:** Chien-Hsin Lu, Pin-Hui Fang, Chih-Hao Lin

**Affiliations:** Department of Emergency Medicine, National Cheng Kung University Hospital, College of Medicine, National Cheng Kung University, 70403, No.138, Shengli Rd., North District, Tainan, Taiwan

**Keywords:** Dispatcher-assisted cardiopulmonary resuscitation, Trauma, Out-of-hospital cardiac arrest

## Abstract

**Background:**

Resuscitation efforts for traumatic patients with out-of-hospital cardiac arrest (OHCA) are not always futile. Dispatcher-assisted cardiopulmonary resuscitation (DA-CPR) during emergency calls could increase the rate of bystander cardiopulmonary resuscitation (CPR) and thus may enhance survival and neurologic outcomes of non-traumatic OHCA. This study aimed to examine the effectiveness of DA-CPR for traumatic OHCA.

**Methods:**

A retrospective cohort study was conducted using an Utstein-style population database with data from January 1, 2014, to December 31, 2016, in Tainan City, Taiwan. Voice recordings of emergency calls were retrospectively retrieved and reviewed. The primary outcome was an achievement of sustained (≥2 h) return of spontaneous circulation (ROSC); the secondary outcomes were prehospital ROSC, ever ROSC, survival at discharge and favourable neurologic status at discharge. Statistical significance was set at a *p*-value of less than 0.05.

**Results:**

A total of 4526 OHCA cases were enrolled. Traumatic OHCA cases (*n* = 560, 12.4%), compared to medical OHCA cases (*n* = 3966, 87.6%), were less likely to have bystander CPR (10.7% vs. 31.7%, *p* < 0.001) and initially shockable rhythms (7.1% vs. 12.5%, *p* < 0.001). Regarding DA-CPR performance, traumatic OHCA cases were less likely to have dispatcher recognition of cardiac arrest (6.3% vs. 42.0%, *p* < 0.001), dispatcher initiation of bystander CPR (5.4% vs. 37.6%, *p* < 0.001), or any dispatcher delivery of CPR instructions (2.7% vs. 20.3%, *p* < 0.001). Stepwise logistic regression analysis showed that witnessed cardiac arrests (aOR 1.70, 95% CI 1.10–2.62; *p* = 0.017) and transportation to level 1 centers (aOR 1.99, 95% CI 1.27–3.13; *p* = 0.003) were significantly associated with achievement of sustained ROSC in traumatic OHCA cases, while DA-CPR-related variables were not (All *p* > 0.05).

**Conclusions:**

DA-CPR was not associated with better outcomes for traumatic OHCA in achieving a sustained ROSC. The DA-CPR program for traumatic OHCAs needs further studies to validate its effectiveness and practicability, especially in the communities where rules for the termination of resuscitation in prehospital settings do not exist.

## Introduction

Out-of-hospital cardiac arrest (OHCA) is a major public health concern. Dispatch centers constitute an important link in the chain of survival for patients with OHCA [1, 2]. Dispatcher-assisted cardiopulmonary resuscitation (DA-CPR) during emergency calls could increase the rate of bystander cardiopulmonary resuscitation (CPR) and thus may enhance survival and neurologic outcomes of non-traumatic OHCA [3–5].

Traumatic OHCA generally has a higher incidence in the young population and is associated with significant mortality and grave neurological outcomes [6, 7]. The resuscitation of traumatic OHCA is broadly considered ineffective; however, these efforts were not always futile [8]. The rate of ever achieving spontaneous return of circulation (ROSC) in traumatic OHCAs, which was up to nearly 30%, could be comparable to that in medical OHCAs [6, 9, 10]. Approximately 10% of traumatic OHCA cases who survived to hospital admission had good neurological outcomes [11]. Survival of traumatic cardiac arrests gradually increased over years [12].

Traumatic OHCA cases, compared to medical OHCA cases, were less likely to receive bystander CPR or have resuscitation commenced by emergency medical technicians (EMTs) [13]. Despite temporal increases in rates of bystander CPR administration and paramedic resuscitation, traumatic OHCA survival remains poor [13]. Although bystander CPR is generally considered an important factor to enhance the outcomes of medical OHCAs [14, 15], the effect of bystander CPR on the outcomes of traumatic OHCAs is questionable [16–18]. The DA-CPR program aims to increase bystander CPR rates for OHCA cases; however, the effect of DA-CPR on traumatic OHCA cases has been examined rarely [19]. This study aimed to 1) describe the patient characteristics and DA-CPR performance in traumatic and medical OHCAs; 2) evaluate the effects of DA-CPR on traumatic OHCAs; and 3) explore the obstacles that impeded DA-CPR in traumatic OHCAs.

## Methods

### Study design and settings

A retrospective cohort study was conducted using an Utstein-style population database in Tainan city, Taiwan. Patients with OHCA who were transported by local emergency medical services (EMS) system between January 1, 2014, and December 31, 2016, were enrolled. Cardiac arrest was defined as the absence of signs of circulation confirmed by EMTs on the scene. Patients with a known pregnancy; who are less than 8 years old; and who have obvious signs of irreversible death, severe hypothermia, or valid do-not-attempt-resuscitation (DNAR) orders were excluded. Patients with evidence of hanging, drowning, electric shocks, or lightning strikes were also excluded [20]. The causes of cardiac arrest were classified as traumatic or medical based on the clinical judgements of the EMS providers and physicians in charge. A traumatic cardiac arrest was defined as a cardiac arrest that was consequent upon a prior traumatic event; those not classified as traumatic were defined as medical. Bystander CPR was defined as an ongoing CPR by bystanders that were confirmed by the first EMT on the scene.

### EMS system in Tainan city

Tainan City constitutes an area of 2192 km^2^ with a population of 1.9 million. The EMS dispatch centre in Tainan is single and centralized. The annual EMS call volume in Tainan was 94,642 in 2016, which is equivalent to 13.8 calls per 100,000 population per day. The averages of EMS response time (interval of ambulance departure from the fire station to ambulance arrival at the scene), the EMS time at the scene (interval of ambulance arrival at the scene to ambulance departure from the scene), and the EMS transport time (interval of ambulance departure from the scene to ambulance arrival at the hospital) were 6.6, 8.0, and 8.2 min, respectively. During the study period, all EMTs performed CPR according to Taiwanese guidelines based on the American Heart Association, European Resuscitation Council, and International Liaison Committee on Resuscitation 2010 Guidelines. Patients with OHCA received CPR for two full cycles (approximately 5 min) before being transported to a designated hospital. Application of an automated external defibrillator was mandatory during resuscitation for medical OHCA but was optional for traumatic OHCA [16]. Resuscitation continued during transport unless ROSC was achieved. Rules for the termination of resuscitation in prehospital settings did not exist. As such, all patients with cardiac arrest who were assessed by EMTs were sent to a hospital unless obvious signs of irreversible death were present [21].

### DA-CPR program

The DA-CPR program in Tainan City was initiated on June 1, 2013. As part of the Pan-Asian Resuscitation Outcome Study Phase II (PAROS-II), Tainan adopted a comprehensive DA-CPR package, which has been described in previous studies [3, 22]. All dispatchers participated in 4-h DA-CPR training course. Dispatchers were trained to comply with a streamlined, two-step question approach to identify possible cardiac arrests. The two-step question consisted of “Is the victim conscious?” and “Is the victim breathing normally?” If the callers responded that the victim was unconscious and was not breathing normally, the presumptive diagnosis was cardiac arrest, and the dispatcher initiated a protocol that delivered instructions for chest compression-only CPR.

Voice recordings of emergency calls for patients with OHCA who were confirmed by EMTs on scene were retrospectively retrieved and reviewed. A team of seven EMTs who received two courses of 4-h evaluation training reviewed the voice recordings every week. The review protocol was consistent with the Singapore version [22]. These reviewing EMTs identified barriers that impeded successful DA-CPR instructions and classified them into one or more of 15 pre-determined barrier types, which were derived from a prior literature review and a focus group discussion in Tainan City, with the provision to enter any unexpected barriers as free text [22]. A medical director then reviewed the results every 2 weeks and randomly double-checked the EMTs’ reviews to ensure quality. A feedback session was conducted monthly where the medical director and dispatchers assembled to discuss the overall performance and the specific DA-CPR cases from the preceding month.

### Characteristics of OHCAs

Data were obtained from a citywide OHCA registry database using paper and computer interfaces. The OHCA registry system was constructed using dispatch registries, EMS run registries, EMS cardiac arrest registries, and an OHCA registry for hospital care and outcomes. Collected data included the information required for the international Utstein-style criteria, which included the patient’s age, gender, witness status, past medical history, EMS response time, EMS time at the scene, EMS transport time, initial cardiac rhythms, presence of bystander CPR, extent and amount of emergency care, achievement of ROSC, hospital admission, and survival and neurologic outcome at discharge [20].

The measurements of DA-CPR consisted of dispatcher recognition of cardiac arrests, dispatcher initiation of bystander CPR, any dispatcher delivery of CPR instructions (defined as the presence of CPR instruction given by dispatchers), and full dispatcher delivery of CPR instructions (defined as good quality of uninterrupted CPR instructions until the arrival of EMTs).

A comparison of traumatic and medical OHCAs was conducted [6, 13]. Categorical variables are shown as numbers and percentages, while quantitative data are shown as mean values and standard deviations (SDs). We used the chi-squared test or Fisher’s exact test for categorical variables and the Mann–Whitney U test for continuous variables when applicable. A two-tailed *p*-value less than 0.05 was considered statistically significant.

The statistical software SPSS (version 17; SPSS Inc., Chicago, IL, USA) was used for statistical analysis.

### Effects of DA-CPR on traumatic OHCAs

The primary outcome evaluated was an achievement of sustained (≥2 h) ROSC [16]. The secondary outcomes were prehospital ROSC, ever ROSC, survival at 24 h, survival at discharge, and good neurological status at discharge defined by the cerebral performance category (CPC) scale I or II [16].

The odds ratio (OR) and its 95% confidence interval (CI) were used as the outcome measures. Univariate analysis was conducted to examine the association of variables and the primary outcome. Multivariate analyses were performed to examine the association and interaction among independent variables and the primary outcome. A stepwise variable selection procedure was applied to obtain the final logistic regression model. Significance levels for entry and to remain were set at 0.15 to avoid the exclusion of potential candidate variables. The final logistic regression model was identified by sequentially excluding individual variables with a *p*-value > 0.05 until all regression coefficients were significant. If the variable of interest, i.e., DA-CPR-related variables, was excluded during the model-fitting process, it was forcibly entered in the final regression model to estimate the effect on the outcomes.

### Obstacles that impeded DA-CPR

The obstacles that impeded DA-CPR for traumatic and medical OHCAs were reviewed and compared [23, 24].

### Ethical consideration

The study was in accordance with ethical standards and was approved by the Institutional Review Board in National Cheng Kung University Hospital (B-ER-107-228).

## Results

### Study objects

**Fig. 1:**
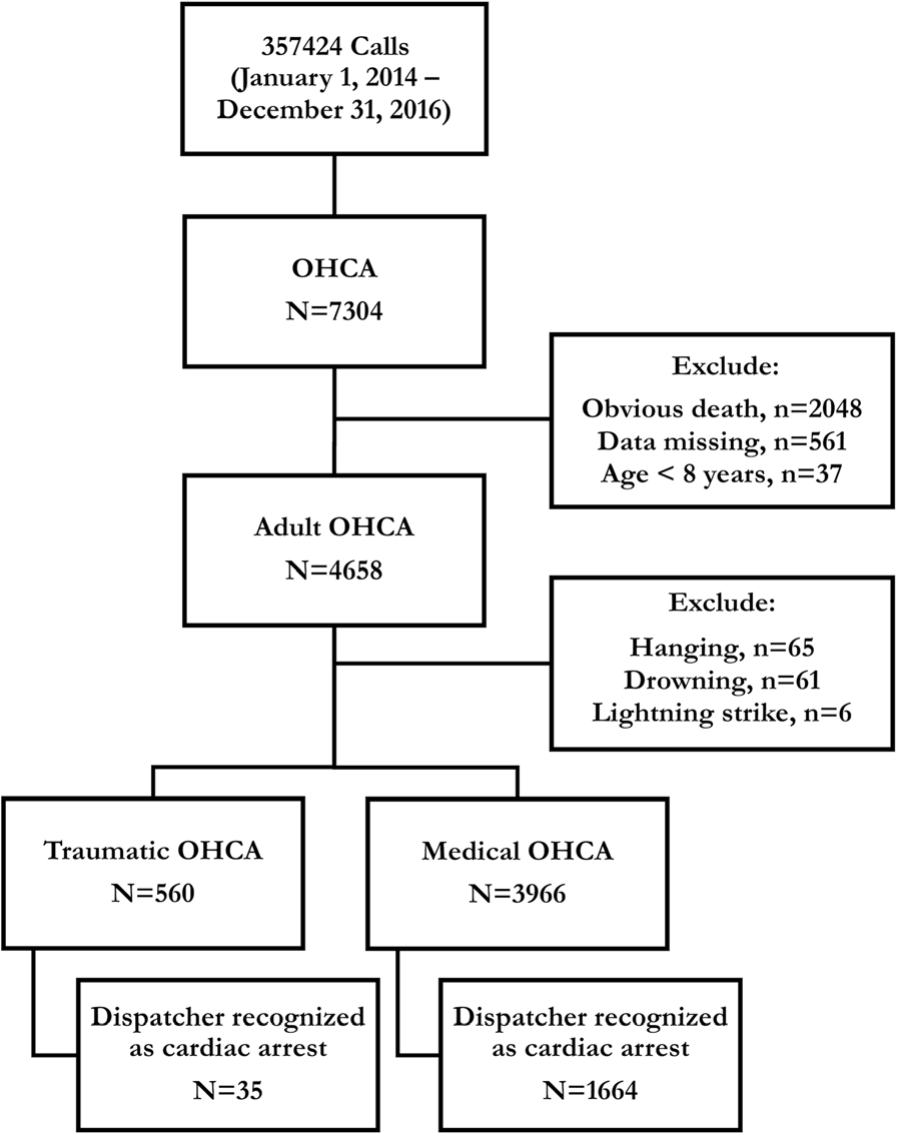
Overview of the patients with out-of-hospital cardiac arrest during the study period. Abbreviation: OHCA, out-of-hospital cardiac arrest

Among the 7304 EMS-assessed OHCA cases during the study period, 4526 cases were included in the analysis after excluding paediatric (< 8 years) patients (*n* = 37), obvious deaths (*n* = 2048), hangings (*n* = 65), drownings (*n* = 61), lightning strikes (*n* = 6), and missing data (*n* = 561). Of the OHCA cases enrolled, 560 (12.4%) were considered traumatic and 3966 (87.6%) were considered medical. Figure 1 provides an overview of OHCA cases evaluated during the study period.

### Characteristics of OHCAs

The demographic findings of OHCA cases are described in Table 1. Compared to medical OHCA cases, traumatic OHCA cases were significantly younger (52.5 ± 19.7 years vs. 69.2 ± 16.6 years, *p* < 0.001) and male-dominant (73.3% vs. 63.3%, *p* < 0.001). Traumatic OHCA cases were more likely to be witnessed by the public (54.4% vs. 9.4%, *p* < 0.001) but less likely to be witnessed by families (18.8% vs. 68.6%, *p* < 0.001). Traumatic OHCA cases were less likely to have bystander CPR (10.7% vs. 31.7%, *p* < 0.001) and initially shockable rhythms (7.1% vs. 12.5%, *p* < 0.001).

**Table 1:** Characteristics of patients with out-of-hospital cardiac arrest

	Traumatic OHCA (*N* = 560)	Medical OHCA (*N* = 3966)	*P* value
Gender (Male)	413 (73.5%)	2530 (63.3%)	< 0.001
Age			
Years, mean (±SD)	52.5 (±19.7)	69.2 (±16.6)	< 0.001
> 55 years (n, %)	255 (45.5%)	3223 (81.3%)	< 0.001
Medical history			
Diabetes	40 (7.1%)	1078 (27.2%)	< 0.001
Hypertension	57 (10.2%)	1438 (36.3%)	< 0.001
Malignancy	10 (1.8%)	469 (11.8%)	< 0.001
COPD/Asthma	16 (2.9%)	300 (7.6%)	< 0.001
Stroke	9 (1.6%)	418 (10.5%)	< 0.001
Liver disease	8 (1.4%)	141 (3.6%)	0.008
Renal disease	14 (2.5%)	449 (11.3%)	< 0.001
Heart disease	33 (5.9%)	902 (22.7%)	< 0.001
Witnessed cardiac arrest	272 (48.6%)	1948 (49.1%)	0.809
By public	148 (54.4%)	184 (9.4%)	< 0.001
By families	51 (18.8%)	1336 (68.6%)	< 0.001
Bystander CPR	60 (10.7%)	1257 (31.7%)	< 0.001
Breathing and chest compression	9 (15.0%)	209 (16.6%)	0.740
Breathing only	3 (5.0%)	16 (1.3%)	0.052
Chest compression only	48 (80.0%)	1032 (82.1%)	0.679
EMS response time (mean ± SD, sec)	434.6 (243.7)	411.2 (202.6)	0.013
EMS scene time (mean ± SD, sec)	658.6 (499.7)	651.4 (370.5)	0.682
EMS transport time (mean ± SD, sec)	457.8 (483.0)	385.8 (420.7)	< 0.001
Prehospital use of laryngeal mask airway	398 (71.1%)	3175 (80.1%)	< 0.001
Prehospital use of epinephrine	18 (3.2%)	173 (4.4%)	0.206
Use of automated external defibrillator	449 (80.2%)	3650 (92.0%)	< 0.001
Shockable rhythms	32 (7.1%)	455 (12.5%)	< 0.001
Sent to Level 1 centers	148 (26.4%)	1043 (26.3%)	0.964
Outcomes			
Prehospital ROSC	20 (3.6%)	182 (4.6%)	0.274
Ever ROSC	131 (23.4%)	897 (22.6%)	0.682
Sustained (≥2 h) ROSC	105 (18.8%)	837 (21.1%)	0.199
Survival at 24 h	75 (13.4%)	660 (16.6%)	0.051
Survival at discharge	11 (2.0%)	181 (4.6%)	0.004
Good neurologic outcome at discharge	4 (0.7%)	110 (2.8%)	0.002

Abbreviations: *OHCA* out-of-hospital cardiac arrest; *SD* standard deviation; *COPD* chronic obstructive pulmonary disease; *CPR* cardiopulmonary resuscitation; *EMS* emergency medical services; *ROSC* return of spontaneous circulation

There were no statistical significances between the traumatic and medical OHCA cases regarding the achievements of prehospital ROSC (3.6% vs. 4.6%), ever ROSC (23.4% vs. 22.6%), sustained (≥2 h) ROSC (18.8% vs. 21.1%), or survival at 24 h (13.4% vs. 16.6%) (All *p* ≥ 0.05). Compared to medical OHCA cases, traumatic OHCA cases were less likely to survive (2.0% vs. 4.6%, *p* = 0.004) and show good neurologic outcomes (0.7% vs. 2.8%, *p* = 0.002) at discharge.

### DA-CPR performance

Table 2 describes DA-CPR performance for OHCA cases. Dispatchers were less likely to comply with dispatch protocols for traumatic OHCA cases (14.6% vs. 59.4%, *p* < 0.001). Traumatic OHCA cases were less likely to have dispatcher recognition of cardiac arrest (6.3% vs. 42.0%), dispatcher initiation of bystander CPR (5.4% vs. 37.6%), or any dispatcher delivery of CPR instructions (2.7% vs. 20.3%) (All *p* < 0.001).

**Table 2:** Dispatcher-assisted cardiopulmonary resuscitation for patients with out-of-hospital cardiac arrest

	Traumatic OHCA (*N* = 560)	Medical OHCA (*N* = 3966)	*P* value
Compliance of dispatch protocol			
Dispatchers evaluate both the consciousness and presence of normal breathing	82 (14.6%)	2355 (59.4%)	< 0.001
Dispatchers only evaluate the consciousness	107 (19.1%)	2642 (66.6%)	< 0.001
Dispatchers only evaluate the presence of normal breathing	99 (17.7%)	2751 (69.4%)	< 0.001
Dispatcher-recognition of cardiac arrests	35 (6.3%)	1664 (42.0%)	< 0.001
Dispatcher-initiation of bystander CPR	30 (5.4%)	1491 (37.6%)	< 0.001
Any dispatcher-delivery of CPR instructions	15 (2.7%)	806 (20.3%)	< 0.001
Full dispatcher-delivery of CPR instructions	1 (0.2%)	40 (1.0%)	0.054

Abbreviation: *CPR* cardiopulmonary resuscitation

### Effects of DA-CPR on traumatic OHCAs

Traumatic OHCA cases that were identified as cardiac arrests by dispatchers (*n* = 35), compared to medical OHCA cases (*n* = 525), had higher rates of bystander CPR (37.1% vs. 9.0%, *p* < 0.001) and more initially shockable rhythms (14.3% vs. 5.1%, *p* = 0.024). There were no significant differences between the two groups regarding any outcome measure (Table 3).

**Table 3:** Characteristics of traumatic patients with out-of-hospital cardiac arrest who were and were not recognized as cardiac arrest by dispatchers

	Dispatcher recognition of OHCA		
	Yes (*N* = 35)	No (*N* = 525)	*P* value
Gender (Male)	24 (68.6%)	388 (73.9%)	0.488
Age			
Years, mean (±SD)	58.6 (±20.1)	51.9 (±19.6)	0.051
> 55 years (n, %)	20 (57.1%)	235 (44.6%)	0.154
Mechanism			1.000
Penetrating injury	0	6 (1.1%)	
Blunt injury	35 (100%)	519 (98.9%)	
Witnessed cardiac arrest	15 (42.9%)	257 (49.0%)	0.485
Bystander CPR	13 (37.1%)	47 (9.0%)	< 0.001
Prehospital use of laryngeal mask airway	24 (68.6%)	374 (71.2%)	0.736
Prehospital use of epinephrine	0	18 (3.4%)	0.619
Use of automated external defibrillator	30 (85.7%)	419 (79.8%)	0.396
Initially shockable rhythms	5 (14.3%)	27 (5.1%)	0.042
Sent to level 1 centers	137 (26.1%)	148 (26.4%)	0.488
Outcomes			
Prehospital ROSC	1 (2.9%)	19 (3.6%)	1.000
Ever ROSC	6 (17.1%)	125 (23.8%)	0.367
Sustained (≥2) ROSC	6 (17.1%)	101 (19.2%)	0.760
Survival at 24 h	4 (11.4%)	72 (13.7%)	0.702
Survival at discharge	1 (2.9%)	10 (1.9%)	0.694
Good neurologic outcomes at discharge	1 (2.9%)	3 (0.6%)	0.120

Abbreviations: *OHCA* out-of-hospital cardiac arrest; *SD* standard deviation; *CPR* cardiopulmonary resuscitation; *ROSC* return of spontaneous circulation

Table 4 shows the effects of key variables on the achievement of sustained (≥2 h) ROSC in traumatic OHCA cases. Dispatcher recognition of cardiac arrests, dispatcher initiation of bystander CPR, any dispatcher delivery of CPR instructions, full dispatcher delivery of CPR instructions, or presence of bystander CPR had statistically insignificant effects in the univariate and multivariate models (all *p* > 0.05). The stepwise logistic regressions showed that witnessed cardiac arrests (adjusted OR [aOR] 1.70, 95% CI 1.10–2.62; *p* = 0.017) and transportation to level 1 centers (aOR 1.99, 95% CI 1.27–3.13; *p* = 0.003) were significantly associated with the achievement of sustained ROSC in traumatic OHCA cases, while DA-CPR-related variables were not (All *p* > 0.05).

**Table 4:** Unadjusted (univariate model) and adjusted (logistic regression model) odds ratios for achieving a sustained (≥2 h) return of spontaneous circulation in traumatic patients with out-of-hospital cardiac arrest

	Unadjusted analysis		Adjusted analysis	
	OR (95% Cls)	*P* value	aOR (95% Cls)	*P* value
Male	0.99 (0.61–1.59)	0.951		
Age (8–55 years)	1.15 (0.75–1.78)	0.528		
Penetrating trauma	2.19 (0.40–12.12)	0.357		
Witnessed cardiac arrest	1.68 (1.09–2.59)	0.017	1.70 (1.10–2.62)	0.017
Dispatcher-recognition of cardiac arrests	0.89 (0.36–2.20)	0.801	1.02 (0.18–5.99)	0.979
Dispatcher-initiation of bystander CPR	0.86 (0.32–2.30)	0.764	0.61 (0.07–5.13)	0.650
Any dispatcher-delivery of CPR instructions	1.09 (0.30–3.92)	0.900	0.80 (0.06–10.81)	0.868
Full dispatcher-delivery of CPR instructions	2.19 (0.40–12.12)	0.357	4.51 (0.29–69.41)	0.280
Bystander CPR	1.09 (0.56–2.14)	0.793		
Prehospital use of epinephrine	1.25 (0.40–3.87)	0.701		
Initially shockable rhythms	1.76 (0.79–3.93)	0.162		
Prehospital use of laryngeal mask airway	0.91 (0.58–1.45)	0.698		
Sent to level 1 centers	1.98 (1.26–3.10)	0.003	1.99 (1.27–3.13)	0.003

Abbreviations: *CIs* confidence intervals; *CPR* cardiopulmonary resuscitation; *OR* odds ratio; *aOR* adjusted odds ratio

The logistic regression analysis showed that none of the DA-CPR-related variables was significantly associated with the secondary outcomes (that is, prehospital ROSC, ever ROSC, survival at 24 h, survival at discharge, or good neurological status at discharge) (All *p* > 0.05).

### Obstacles that impeded DA-CPR

Patients with OHCA that did not receive full dispatcher delivery of CPR instructions were eligible for further analysis to identify the obstacles that impeded DA-CPR (Table 5). The obstacles in traumatic OHCA cases (*n* = 559), compared to medical OHCA cases (*n* = 3926), were more likely associated with “callers physically distant from the victims” (34.0% vs. 10.0%), “difficulty of access to the victims” (30.1% vs. 19.4%;), “third party callers such as policepersons” (11.1% vs. 2.3%), or “dangerous scenes” (5.2% vs. 0.2%) (All *p* < 0.001).

**Table 5:** Analysis of obstacles that impeded dispatcher-assisted cardiopulmonary resuscitation for patients with out-of-hospital cardiac arrest

	Traumatic OHCA (*N* = 559)	Medical OHCA (*N* = 3926)	*P* value
Caller not beside the victim	340 (60.8%)	1082 (27.6%)	< 0.001
Caller not physically alongside the victim	190 (34.0%)	393 (10.0%)	< 0.001
Difficult access to the victim	168 (30.1%)	760 (19.4%)	< 0.001
Third party caller	62 (11.1%)	90 (2.3%)	< 0.001
Dangerous scene	29 (5.2%)	9 (0.2%)	< 0.001
Dispatcher can’t recognize the need for CPR	253 (45.3%)	1306 (33.3%)	< 0.001
Caller hanged up	16 (2.9%)	124 (3.2%)	0.706
Overly distraught	15 (2.7%)	124 (3.2%)	0.544
Dispatcher hanged up	12 (2.1%)	74 (1.9%)	0.673
Patient’s status changed	7 (1.3%)	97 (2.5%)	0.073
Caller is unable to perform CPR	4 (0.7%)	12 (0.3%)	0.129
Caller is unable to move the patient	4 (0.7%)	2 (0.1%)	0.003
CPR is already ongoing	2 (0.4%)	14 (0.4%)	1.000
Caller refused to perform CPR	1 (0.2%)	6 (0.2%)	0.606
Valid consent of Do-Not-Attempt-Resuscitate orders	0	0	–

*Abbreviations*: *OHCA* out-of-hospital cardiac arrest, *CPR* cardiopulmonary resuscitation

## Discussion

The results of our study found that the compliance with and performance of DA-CPR for traumatic OHCA cases were poor. The rates of dispatcher recognition of cardiac arrest, dispatcher initiation of bystander CPR, or any dispatcher delivery of CPR instructions were not associated with the achievement of sustained ROSC in traumatic OHCA cases.

Our findings may imply that the concept of chain of survival in traumatic OHCA could be different from that in medical OHCA. In general, medical OHCA events that occur in public locations are more likely to receive bystander CPR than are those in non-public locations [25]. Patients with medical OHCA may receive more resuscitation when they are witnessed by non-family bystanders than by family members [26]. Traumatic OHCA events, compared to medical OHCA events, were more likely to occur in public locations; however, the rate of bystander CPR for traumatic OHCA cases was extremely low. The 119 callers for traumatic OHCA events that occurred in public locations were often not physically alongside the patients [22, 27, 28]. The dispatchers could ask the callers to move beside the victim and redial emergency calls by a mobile phone [29]. However, the bystanders may call 119 after they have already left the scene and may hesitate when asked to approach the traumatic patient. Concerns of bloodborne diseases and safety issues in trauma scenes could impede the bystanders from providing a physical assessment and even resuscitation efforts [30].

The bystander CPR rate could significantly increase if dispatchers could recognize the need to perform CPR on the patient, even in traumatic OHCA cases. Compared with those not recognized by dispatchers, traumatic OHCA cases that were recognized by dispatchers had more bystander CPR and more initially shockable rhythms. We were unable to evaluate the characteristics of the bystanders. Their willingness to stay alongside the traumatic patients with OHCA and to perform resuscitation at the scene is an important issue to investigate. We suspect that the degree of chaos at the trauma scene and the apparent severity of injury could influence the likelihood of bystander CPR.

The compliance of DA-CPR protocols was generally poor when the dispatchers evaluated the calls regarding traumatic patients. Severe traumatic incidents may give dispatchers the impression that the prognosis of victims with traumatic OHCAs will be grave even when resuscitation efforts are performed, which may consequently result in poor compliance with DA-CPR protocols and thus lower the recognition rate of cardiac arrest.

The reason that dispatchers were unable to correctly identify cardia arrest in traumatic cases deserves further exploration. In this study, the dispatchers used the same protocol to identify possible cardiac arrests in either traumatic or medical patients. The protocol consisted of a streamlined, two-step question (that is, “Is the victim conscious?” and “Is the victim breathing normally?”) Although the two-step question performs well in identifying medical OHCA [3, 22], its practicability and effectiveness to identify cardiac arrest in traumatic patients are rarely examined [19]. We suspected that the optimal protocol for dispatchers to identify cardiac arrest in traumatic patients could be different from that in medical patients.

Once the dispatchers recognized an event of OHCA in emergency calls, the rate of initiating and executing CPR instructions for traumatic OHCA patients and medical OHCA patients were similar. We did not evaluate the personality characteristics or telephone skills of individual dispatchers. We assumed that the dispatchers who recognized cardiac arrests in traumatic patients were more aggressive in providing resuscitation efforts, which may have thus resulted in a type 1 error with better outcomes for traumatic OHCA patients. However, the study results did not find improved outcomes in the group.

The policy of “resuscitation during transportation” is not common in Western EMS systems. However, due to the lack of rules for termination of resuscitation in the prehospital settings, many Asian EMS systems utilize the protocols of resuscitation during transportation [21]. A recent study found that high quality CPR metrics (including chest compression fraction, compression rate, and compression depth) were similar at the scene and during ambulance transportation [31]. High quality CPR can be performed by prehospital providers regardless of location [31]. The evolution of mechanical CPR and other devices could have impacts on current EMS protocols of managing OHCAs [32].

Our study results showed that bystander CPR, initially shockable rhythms and advanced life supports, such as the use of epinephrine or an advanced airway, were not significantly associated with the achievement of sustained ROSC. We only found that witnessed cardiac arrests and transportation to level 1 centers were significantly associated with the primary outcome. These findings imply that the traditional “load and go” model for traumatic patients might strategically apply to patients with traumatic OHCA as well [13, 16, 33]. The concept of “chain of survival” for traumatic OHCA patients deserves further exploration.

This study had several limitations. First, due to the low crime rate and strict weapon control policy, patients with traumatic OHCA who had penetrative injuries, such as stabbings or gunshots, were extremely rare in the city during the study period. Thus, our study findings might mainly apply in patients with blunt trauma. Second, this observational study was conducted in a setting with suboptimal compliance of DA-CPR protocols for traumatic OHCA cases. Although factors were adjusted using multivariable analysis, other confounding factors might exist given that this was not a randomized controlled trial. Third, we were unable to validate the actual performance and measure the quality of bystander CPR through the voice recordings in dispatch centers. This technical difficulty may require a more comprehensive system using emergency calls with real-time video communication in the future. Fourth, we were unable to approach the treatment and management of patients in receiving hospitals. The management in individual hospitals might be associated with patient outcomes. Potential limitations could also exist in multi-site studies regarding data integrity, validity, and ascertainment bias. In attempts to minimize these potential sources of bias, this population-based cohort study utilized the time synchronization process, consistent definitions, uniform data collection, and a large sample size. Finally, this study was conducted in an EMS system that utilizes the policy of “resuscitation during transportation” for OHCAs. Thus, the application of our study results should be tailored to local EMS practices.

## Conclusion

Our study results found that DA-CPR was not associated with better outcomes for traumatic OHCAs in achieving a short-term sustained ROSC. The DA-CPR program for traumatic OHCAs needs further studies to validate its effectiveness and practicability, especially in the communities where rules for the termination of resuscitation in prehospital settings do not exist.

## Data Availability

The datasets used and/or analyzed during the current study are available from the corresponding author on reasonable request.

## Abbreviations

CI: Confidence interval
CPC: Cerebral performance category
CPR: Cardiopulmonary resuscitation
DA-CPR: Dispatcher-assisted cardiopulmonary resuscitation
DNAR: Do-not-attempt-resuscitation
EMS: Emergency medical service
EMT: Emergency medical technician
OHCA: Out-of-hospital cardiac arrest
OR: Odds ratio
PAROS-II: Pan-Asian Resuscitation Outcome Study Phase II
ROSC: Return to spontaneous circulation
SD: Standard deviation
